# Genome-Wide DNA Methylation and LncRNA-Associated DNA Methylation in Metformin-Treated and -Untreated Diabetes

**DOI:** 10.3390/epigenomes4030019

**Published:** 2020-09-01

**Authors:** Wendy L. Solomon, Stanton B. E. Hector, Shanel Raghubeer, Rajiv T. Erasmus, Andre P. Kengne, Tandi E. Matsha

**Affiliations:** 1 SAMRC/CPUT/Cardiometabolic Health Research Unit, Department of Biomedical Sciences, Faculty of Health and Wellness Science, Cape Peninsula University of Technology, P.O. Box 1906, Bellville, Cape Town 7530, South Africa; solomonw@cput.ac.za (W.L.S.); hectors@cput.ac.za (S.B.E.H.); shanelraghubeer@gmail.com (S.R.); 2 Division of Chemical Pathology, Faculty of Health Sciences, National Health Laboratory Service (NHLS) and University of Stellenbosch, Cape Town 7505, South Africa; rte@sun.ac.za; 3 Non-Communicable Diseases Research Unit, South African Medical Research Council, Cape Town 7505, South Africa; andre.kengne@mrc.ac.za; 4 Department of Medicine, University of Cape Town, Cape Town 7700, South Africa

**Keywords:** metformin, DNA methylation, lncRNA, diabetes mellitus, Africa

## Abstract

Metformin, which is used as a first line treatment for type 2 diabetes mellitus (T2DM), has been shown to affect epigenetic patterns. In this study, we investigated the DNA methylation and potential lncRNA modifications in metformin-treated and newly diagnosed adults with T2DM. Genome-wide DNA methylation and lncRNA analysis were performed from the peripheral blood of 12 screen-detected and 12 metformin-treated T2DM individuals followed by gene ontology (GO) and KEGG pathway analysis. Differentially methylated regions (DMRs) observed showed 22 hypermethylated and 11 hypomethylated DMRs between individuals on metformin compared to screen-detected subjects. Amongst the hypomethylated DMR regions were the SLC gene family, specifically, SLC25A35 and SLC28A1. Fifty-seven lncRNA-associated DNA methylation regions included the mitochondrial ATP synthase-coupling factor 6 (ATP5J). Functional gene mapping and pathway analysis identified regions in the axon initial segment (AIS), node of Ranvier, cell periphery, cleavage furrow, cell surface furrow, and stress fiber. In conclusion, our study has identified a number of DMRs and lncRNA-associated DNA methylation regions in metformin-treated T2DM that are potential targets for therapeutic monitoring in patients with diabetes.

## 1. Introduction

DNA methylation, the most widely studied epigenetic mechanism, involves the covalent addition of a methyl group at the 5′ position of the cytosine ring within the 5′-CpG-3′ dinucleotides to create a 5-methylcytosine (5-mC). The target of DNA methylation, catalyzed by DNA methyltransferases (DNMTs) enzymes, are CpG nucleotides, which are usually unmethylated [1]. These CpG nucleotides occur at high-frequency in the promoter regions of genes and are frequently associated with hyper- or hypomethylation events [2]. Hypermethylation of promoter CpG islands can result in suppression of gene expression, whereas hypomethylation is associated with the transcriptional activation of affected genes [3]. Various studies suggest that these modifications may alter the transcriptional activity of genes and contribute to pathogenic conditions, such as the type 2 diabetes mellitus (T2DM) phenotype [4,5]. Studies also indicate that response to anti-diabetic agents and occurrence of diabetes complications can result from the actions of DNA methylation [4,6]. 

Although progression of disease cannot solely be attributed to DNA methylation, the impact of long non-coding RNAs (lncRNAs) on biological and pathologic processes have also been linked to various conditions including cancers and metabolic diseases [7,8]. LncRNAs are transcription products greater than 200 nucleotides with limited protein coding function [9]. They have been implicated in the regulation gene expression at the epigenetic, transcriptional, and post-transcription level [10]. Studies show that lncRNAs may also play a role in the diagnosis and therapeutic management of diabetes due to their involvement in regulatory processes and complications of T2DM [11,12].

Metformin, a drug commonly used for the treatment of T2DM, is highly effective with minimal side effects [13]. It has the ability to promote the phosphorylation and activation of AMP-activated protein kinase (AMPK), which results in the inhibition of gluconeogenic genes. In addition to glucose metabolism, the activation of AMPK impacts other pathways, such as lipid metabolism, mitochondrial biogenesis, autophagy, cell growth, and circadian rhythm [14]. Once activated, AMPK phosphorylates epigenetic enzymes, such as DNA methyltransferases (DNMTs), resulting in their inhibition [15]. The effects of metformin on DNA methylation include both hypo- and hypermethylation at the promoters of different genes, which in turn, could act to enhance or suppress gene expression [16,17,18,19]. Metformin was shown to affect DNA methylation even in healthy individuals immediately 10 h after drug administration [19]. These alterations in DNA methylation has also been evident in cancer related studies, showing that DNA methylation plays a role in the antidiabetic and potential anti-cancer actions of metformin [15,20,21,22].

Despite recent advances in the role of DNA methylation and diabetes, data on its effect in those under treatment with metformin in Africa are lacking. We, therefore, aimed to characterize the DNA methylation modifications in newly diagnosed and metformin-treated South Africans with T2DM. The knowledge gained could be used as a basis for further studies to elucidate the role of DNA methylation in the monitoring and treatment of T2DM within a South African context.

## 2. Results

### 2.1. Clinical Characteristics of the Study Population

The general clinical characteristics of the study population are summarized in Table 1. The study sample comprised 24 participants—12 screen-detected and 12 metformin-treated T2DM. There were no significant differences between the two groups in all the clinical characteristics. The duration of disease in the metformin-treated T2DM ranged from 0.5 to 17 with an average of 5.2 years. 

### 2.2. Differentially Methylated Regions, LncRNA-Associated DNA Methylation, Gene Ontology (GO) and Pathway Analysis

**Figure 1:**
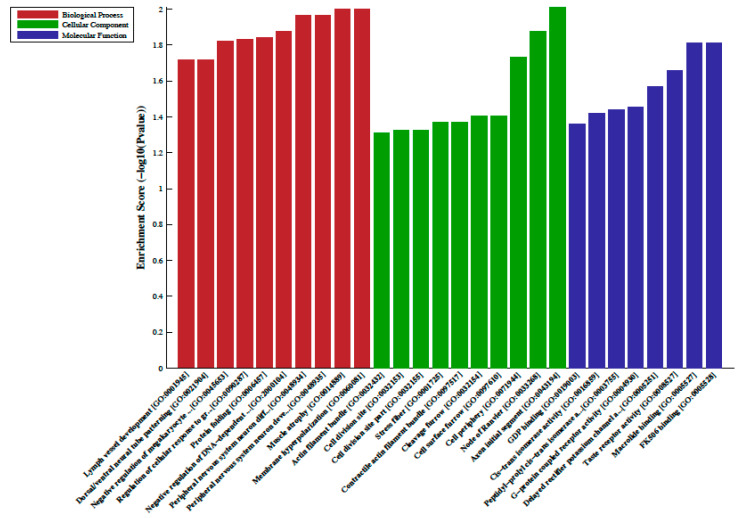
Gene ontology (GO) enrichment analysis of the differentially hypermethylated genes in metformin-treated diabetes. The bar plot shows the top ten enrichment score values of the significant enrichment terms. Enriched GO terms were categorized into biological processes, cellular components, and molecular function. Data are presented as enriched scores expressed as −log10 (*p* value).

**Figure 2:**
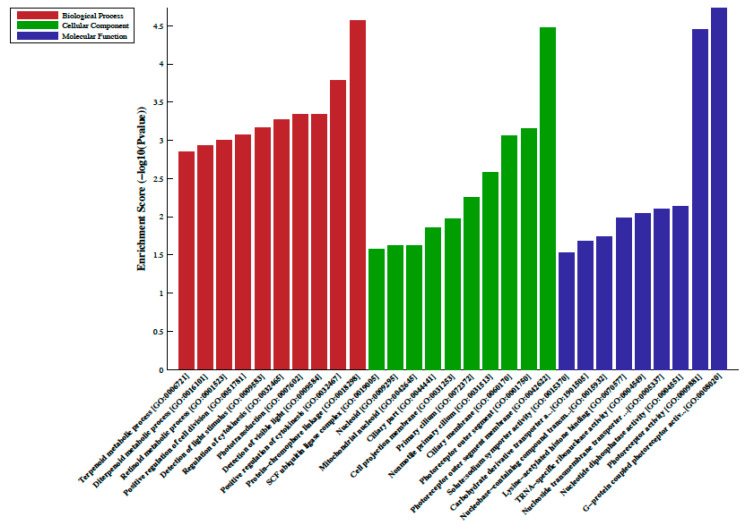
Gene ontology (GO) enrichment analysis of the differentially hypomethylated genes in metformin-treated diabetes. The bar plot shows the top ten enrichment score values of the significant enrichment terms. Enriched GO terms were categorized into biological processes, cellular components, and molecular function. Data are presented as enriched scores expressed as −log10 (*p* value).

A total of 33 differentially methylated regions (DMRs) were observed between individuals on metformin treatment compared to screen-detected subjects. Of these, 22 were hypermethylated, whilst another 11 were hypomethylated in participants treated with metformin, and these are summarized in Table 2. Lnc-associated DNA methylation peaks in the promoter regions are summarized in Table 3 showing that 36 were hypermethylated, and 21 were hypomethylated in individuals on metformin. KEGG pathway analysis revealed no enriched pathways. Based on GO analyses, we retrieved the biological process, cellular process, and molecular function of the DMRs, and these are presented in Figure 1 and Figure 2. The top enrichment scores for cellular processes of hypermethylated DMRs in subjects on metformin were associated with the axon initial segment, node of Ranvier, cell periphery, cleavage furrow, cell surface furrow, and stress fiber (Figure 1), whilst the hypomethylated biological processes were associated with photoreceptor outer segment (Figure 2).

## 3. Discussion

In this study, we measured DNA methylation in diabetic individuals on metformin treatment compared to newly diagnosed diabetes cases and found 33 differentially methylated regions (DMRs) of which 22 (67%) were hypermethylated in diabetes subjects on metformin therapy. Furthermore, 57 lncRNA-associated DNA Methylation regions (36 hypermethylated and 21 hypomethylated) were detected of which 63% were hypermethylated in metformin-treated subjects. Functional pathway analysis of these DMRs revealed that they affect gene expression in the axon initial segment (AIS), node of Ranvier, cell periphery, cleavage furrow, cell surface furrow, and stress fiber.

Amongst the hypomethylated DMRs found in this study were genes in the SLC family, specifically SLC25A35 and SLC28A1. The SLC family is known for its importance in drug development, and their proteins include passive transporters, symporters, and antiporters and are located in cellular and organelle membranes [23]. Transporters facilitate the movement of a specific substrate across the membrane with or against its concentration gradient and sequence analysis of SLC25A35 indicates that it likely functions as an oxaloacetate carrier, implying mitochondrial association [24]. On the other hand, SLC28A1, a high-affinity pyrimidine nucleoside transporter, plays a role in renal reabsorption and has been observed to be impaired during diabetes [25]. Metformin treatment has been associated with lower methylation levels in SLC transporter genes, as was shown in a study conducted on metformin transporter genes in liver tissue [18]. Mitochondrial dysfunction due to diabetes affects oxidative phosphorylation and decreases ATP production. As SLC proteins transport various solutes across the mitochondrial membrane in order to partake in a number of metabolic pathways [26,27], the decrease in methylation and subsequent increase in gene expression of SLC transporters could be indicative of the antidiabetic effect of metformin treatment. It is, therefore, likely that metformin in its demethylation action of SLC mitochondrial carriers could possibly aid cell repair in these patients, however, this requires further investigation.

Functional pathway analysis observed in this study is consistent with the basic pathological abnormalities in Diabetic Peripheral Neuropathy (DPN), such as axonal degeneration and demyelination, lack of sensation, numbness, paresthesia, and allodynia experienced by diabetic individuals [28]. Cell death of nerves in DPN results from multifactorial metabolic imbalances associated with diabetes. The resulting mitochondrial dysfunction through a series of cascade effects involving AMP-activated protein kinase (AMPK), sirtuin (SIRT), and peroxisome proliferator-activated receptor-γ coactivator α (PGCα) suppresses mitochondrial oxidative phosphorylation, resulting in neuronal and axonal degeneration through increased oxidative injury [29,30]. 

Treatment with metformin was shown to decrease the incidence of DPN as was observed by the Bypass Angioplasty Revascularization Investigation 2 Diabetes trial [31]. Although metformin cannot reverse the nerve damage caused by diabetes, it could assist in managing blood glucose levels and improving the symptoms for patients.

In addition to DMRs, 57 lncRNA-associated DNA Methylation Peaks were detected when comparing known diabetic individuals to screen detected patients. Most recently the NONCODE database has updated the numbers of human lncRNAs to 167,150 with numbers still increasing [32]. Recent genome-wide association studies (GWAS) have shown positive correlation of some lncRNAs and diabetes [33]. In a related study, Sathishkumar et al. (2018) found increased levels of lncRNAs in T2DM patients, including HOTAIR, MEG3, LET, MALAT1, MIAT, CDKN2BAS1/ANRIL, XIST, PANDA, GAS5, Linc-p21, ENST00000550337.1, PLUTO, NBR2THRIL, and SALRNA1. The majority of these lncRNAs were involved in cell cycle regulation and senescence with their expression levels correlating to poor glycemic control, insulin resistance, and inflammation [11]. Similarly, HECTD4 and MBTPS1 were identified as the target genes for lncRNAs ENST00000364558 and ENST00000565382, respectively, with involvement in the development of T2DM by means of the lysosome and phagocytic signaling pathways [34]. Our findings indicate several novel lncRNA, including a lncRNA associated with the mitochondrial ATP synthase-coupling factor 6 (ATP5J) enzyme thought to be involved in the oxidative phosphorylation pathway [35]. Our data suggest higher methylation levels of this lncRNA in metformin-treated subjects, possibly pointing to suppression of this lncRNA allowing for ATP5J expression. Although little association was found between metformin and lncRNAs in our study, the significant novel lncRNA identified warrants further investigation to explore possible roles in type 2 diabetes.

The limitations of this study include the small sample size and the inclusion of women only; however, this allowed comparison and limited error that may result in statistical manipulation of a small sample size by sex. Furthermore, we used peripheral blood DNA to perform the genome-wide DNA methylation analysis. Epigenetic changes are believed to be organ specific; however, investigations on peripheral blood DNA have shown consistent methylation patterns with other organs [36,37]. Although the average (5.2 years) duration of disease in metformin-treated subjects was within the four to six years in which a person may have had the condition before clinical diagnosis [38], these findings should be interpreted with caution. In conclusion, our study has identified a number of DMRs and lncRNA-associated DNA methylation regions in metformin-treated T2DM that are potential targets for therapeutic monitoring in diabetes patients. However, these findings require further longitudinal study investigations that can clearly ascertain that these observations are not confounded by the duration and severity of diabetes.

## 4. Materials and Methods

### 4.1. Ethical Approval of the Study

This investigation used data from the Cape Town Vascular and Metabolic Health (VMH) study), which were approved by the Research Ethics Committees of the Cape Peninsula University of Technology and Stellenbosch University (resp., NHREC: REC-230 408-014 and N14/01/003; approved date: 21 May 2018). The Code of Ethics of the World Medical Association (Declaration of Helsinki) was also applied to the study. Signed written consent was obtained from all participants after all procedures were explained in the language of their choice.

### 4.2. Study Procedures

In this case-control study, the participants were females matched for both age and body mass index. All study participants underwent a standardized interview, blood pressure, and anthropometric measurements. A 75 g oral glucose tolerance test (OGTT) was performed on participants with no previous diagnosis of diabetes mellitus. Participants who met the World Health Organisation (WHO) criteria for diabetes were termed as screen-detected or newly diagnosed diabetes. Biochemical parameters analyzed at an ISO 15189 accredited pathology practice (PathCare, Reference Laboratory, Cape Town, South Africa) included the following: plasma glucose, serum insulin, serum creatinine, total cholesterol (TC), high-density lipoprotein cholesterol (HDL-c), triglycerides (TG), low-density lipoprotein cholesterol (LDL), C-reactive protein (CRP), γ-glutamyl transferase (GGT), AST, ALT, and glycated hemoglobin (HbA1c), certified by the National Glycohemoglobin Standardization Program (NGSP). In addition, a full blood count was also done for all participants, and ethylenediaminetetraacetic acid (EDTA) treated blood samples were stored at −20 degrees Celsius for DNA extraction and analysis.

### 4.3. Genome-Wide DNA Methylation Sequencing

Genomic DNA was extracted from peripheral blood using the Wizard Genomic DNA Purification Kit (Promega, Madison, WI, USA) according to the manufacturer’s instructions. At least 2 µg of DNA (concentrations ranging between 70 and 130 ng/μL) with A260/A280 and A260/A230 ratios ≥ 1.8 was shipped frozen on dry ice, as instructed by Arraystar Inc. (Rockville, MD, USA). Methylated DNA immunoprecipitation (MeDIP) was performed by Arraystar Inc. (Rockville, MD, USA) according to Down et al. [39], with minor modifications as follows. About 1 μg of fragmented DNA was prepared for Illumina HiSeq 4000 sequencing as the following steps: (1) end repair of DNA samples with T4 DNA polymerase, Klenow DNA polymerase, and T4 PNK; (2) a single ‘A’ base was added to the 3’ ends with Klenow (exo minus) polymerase; (3) Illumina’s genomic adapters were ligated to DNA fragments; (4) DNA fragments were immunoprecipitated by anti-5-methylcytosine antibody (Diagenode); (5) immunoprecipitated DNA fragments were amplified by PCR amplification; (6) ~300–600 bp DNA fragments were extracted by gel purification. The completed libraries were quantified by Agilent 2100 Bioanalyzer (Agilent Technologies, Santa Clara, CA, USA). The libraries were denatured with 0.1 M NaOH to generate single-stranded DNA molecules, captured on Illumina flow cell, amplified in situ. The libraries were then sequenced on the Illumina HiSeq 4000 following the TruSeq SBS Kit v5 protocol. The enrichment of DNA immunoprecipitation was analyzed by qPCR using specific methylated sites at H19 locus and non-methylated sites at GAPDH.

### 4.4. MeDIP-Seq Data Analysis

The enrichment of DNA immunoprecipitation was analyzed by qPCR using specific methylated sites at H19 locus and non-methylated sites at GAPDH. Image analysis and base calling were performed using Off-Line Basecaller software (OLB V1.8). After passing a Solexa CHASTITY quality filter, the clean reads were aligned to the human genome (UCSC HG19) using HISAT2 software (V2.1.0). Briefly, individual bases generated from original image files have quality scores, which reflect the probability whether base calling is correct or not. The score is calculated by CHASTITY Formula. The CHASTITY (C) of each base in the short reads is determined by the intensity of four colors (IA, IC, IG, and IT here), and the formula means “the ratio of the highest (IC here) of the four (base type) intensities to the sum of highest two (IC and IG here).” The CHASTITY (C) should be no less than 0.6 in the first 25 bases. Statistically significant MeDIP-enriched regions (peaks) detected by MACS v2 were identified by comparison to input background, using a *q*-value threshold of 10^−5^. The peaks in samples were annotated by the nearest gene using the newest UCSC RefSeq database. Differentially methylated regions (DMRs) located within gene promoters (TSS − 2000 bp, TSS + 2000 bp) with statistical significance between the two groups were identified by diffReps (Cut-off: log2FC = 1.0, *p*-value = 10^−4^). 

### 4.5. Gene Ontology (GO) and KEGG Pathway Analysis

The ontology covers three domains, namely biological process, cellular component, and molecular function. Fisher’s exact test was used to determine whether there was more overlap between the DE list and the GO annotation list than would be expected by chance. The *p* value denotes the significance of GO terms enrichment in the DE genes. The lower the *p* value, the more significant the GO term; a *p* value ≤ 0.05 was considered significant. Annotation was performed using standard workflow according to http://geneontology.org/. Pathway analysis was done using the Kyoto Encyclopedia of Genes and Genomes (KEGG) database. The *p* value (EASE score, Fisher’s *p*-value, or hypergeometric *p*-value) denotes the significance of the pathway correlated to the conditions. The lower the *p* value is, the more significant the pathway is; a *p* value ≤ 0.05 was considered significant. 

## Figures and Tables

**Table 1:** Clinical characteristics of the study population.

Characteristics	Screen-Detected Diabetes Mellitus *n* = 12	Known Diabetes Mellitus *n* = 12	*p*-Value
	Mean ± SD	Mean ± SD	
Age (years)	54.8 ± 7.5	53.2 ± 9.6	0.658
Body mass index (kg/m^2^)	33.5 ± 8.9	29.4 ± 5.0	0.174
Waist circumference (cm)	101.3 ± 19.7	91.7 ± 10.5	0.150
Hip circumference (cm)	109.4 ± 16.6	103.3 ± 11.9	0.311
Waist hip ratio	0.92 ± 0.07	0.89 ± 0.05	0.195
Systolic blood pressure (mmHg)	142.9 ± 32.9	136.3 ± 25.9	0.587
Diastolic blood pressure (mmHg)	94.2 ± 22.1	83.8 ± 11.5	0.165
Fasting plasma glucose (mmol/L)	9.1 ± 3.6	11.0 ± 5.8	0.352
Post 2-h plasma glucose (mmol/L)	16.5 ± 4.71	-	-
HbA1c (%)	7.97 ± 2.58	9.33 ± 3.04	0.254
Fasting serum insulin (mIU/L)	15.3 ± 10.6	11.3 ± 7.6	0.316
Triglycerides (mmol/L)	2.18 ± 1.35	2.02 ± 0.96	0.760
Total cholesterol (mmol/L)	6.36 ± 0.85	6.10 ± 1.47	0.607
Low-density lipoprotein-cholesterol (mmol/L)	4.17 ± 0.84	4.08 ± 1.22	0.831
High-density lipoprotein-cholesterol (mmol/L)	1.38 ± 0.58	1.23 ± 0.35	0.448
Ultrasensitive C-reactive protein (mg/L)	11.1 ± 12.3	14.4 ± 12.5	0.531
Serum cotinine (ng/mL)	127.4 ± 149.5	120.7 ± 179.7	0.921
Gamma-glutamyl transferase (IU/L)	64.5 ± 57.4	43.7 ± 27.2	0.287

**Table 2:** Differentially methylated regions (DMRs) in T2DM on metformin versus newly diagnosed cases.

Hypermethylated DMRs					
Gene Name	Genomic Coordinates	DMR Length	log_2_FC	*p*-Value	*q*-Value
XAGE1E	chrX:52260741-52261020	279	1.66	<0.001	0.001
XAGE1B	chrX:52260741-52261020	279	1.66	<0.001	0.001
KIAA1467	chr12:13198981-13199200	219	1.62	<0.001	0.001
ASB2	chr14:94442921-94443160	239	1.6	<0.001	0.001
GABPA	chr21:27105221-27105420	199	1.56	<0.001	0.004
ZNF346	chr5:176448161-176448360	199	1.47	<0.001	0.004
FKBP8	chr19:18655321-18655520	199	1.4	<0.001	0.001
CTAGE15	chr7:143268761-143269080	319	1.38	<0.001	0.001
VIPR1	chr3:42531141-42531360	219	1.37	<0.001	0.026
TMEM204	chr16:1584901-1585100	199	1.31	<0.001	0.002
RNF103-CHMP3	chr2:86948981-86949200	219	1.31	<0.001	0.010
PARVB	chr22:44394581-44394780	199	1.26	<0.001	0.004
POTED	chr21:14980641-14980880	239	1.23	<0.001	0.001
STAG2	chrX:123095761-123095980	219	1.21	<0.001	0.008
KCNQ3	chr8:133459461-133459680	219	1.19	<0.001	0.026
TBCE	chr1:235532261-235532520	259	1.16	<0.001	0.001
GAREML	chr2:26393901-26394160	259	1.14	<0.001	0.003
SEPT12	chr16:4838741-4839000	259	1.13	<0.001	0.003
OR6C3	chr12:55727101-55727440	339	1.11	<0.001	0.011
PPP1R32	chr11:61247661-61247920	259	1.1	<0.001	0.003
ZNF169	chr9:97023241-97023440	199	1.07	<0.001	0.024
TAS1R1	chr1:6616841-6617200	359	1.07	<0.001	0.001
**Hypomethylated DMRs**					
TPD52L2	chr20:62497561-62497920	359	−1	<0.001	0.003
GAGE7	chrX:49217161-49217580	419	−1.15	<0.001	0.011
NUDT10	chrX:51075781-51076040	259	−1.37	<0.001	0.001
OPN1MW2	chrX:153446941-153447140	199	−1.39	<0.001	0.008
OPN1MW	chrX:153446941-153447140	199	−1.39	<0.001	0.008
BRDT	chr1:92415321-92415520	199	−1.39	<0.001	0.023
ELAC2	chr17:12919641-12919840	199	−1.45	<0.001	0.012
SLC25A35	chr17:8196461-8196680	219	−1.52	<0.001	0.004
C18orf8	chr18:21081741-21081940	199	−1.55	<0.001	0.002
SLC28A1	chr15:85429461-85429660	199	−1.67	<0.001	0.008
FBXW8	chr12:117350081-117350320	239	−1.79	<0.001	0.008

Gene name refers to the name of the DMR-associated gene. Genomic coordinates refers to the genomic locus of the DMR. DMR Length refers to the length of the DMR. log2FC refers to the fold change of normalized tag counts between two groups (log2 transformed). The *p*-value refers to the *p*-value of the DMR, the smaller, the more significant. The *q*-value refers to the Benjamini-Hochberg False Discovery Rate (BH FDR) corrected *p*-value.

**Table 3:** LncRNA-associated DNA methylation peaks of known diabetes versus screen-detected diabetes.

Hypermethylated					
Gene Name	Genomic Coordinates	DMR Length	log2FC	*p*-Value	*q*-Value
SLC26A9	chr1:205895421-205895620	199	1.91	<0.001	0.001
FAM223A	chrX:153859601-153859800	199	1.82	<0.001	0.001
SDK2	chr17:71432461-71432680	219	1.69	<0.001	0.001
XAGE1B	chrX:52260741-52261020	279	1.66	<0.001	0.001
SCRIB	chr8:144877441-144877660	219	1.62	<0.001	0.001
KIAA1467	chr12:13198981-13199200	219	1.62	<0.001	0.001
AK092098	chr11:63591421-63591720	299	1.58	<0.001	0.001
ATP5J	chr21:27105221-27105420	199	1.56	<0.001	0.004
AF420437	chr1:146216561-146217120	559	1.49	<0.001	0.002
ZNF346	chr5:176448161-176448360	199	1.47	<0.001	0.004
AX747590	chr8:12435501-12435760	259	1.46	<0.001	0.001
AK128525	chr2:89160101-89160340	239	1.45	<0.001	0.001
XLOC_007349	chr9:38128521-38128740	219	1.4	<0.001	0.001
FKBP8	chr19:18655321-18655520	199	1.4	<0.001	0.001
LOC101927468	chr1:147717321-147717520	199	1.38	<0.001	0.001
CTAGE15	chr7:143268761-143269080	319	1.38	<0.001	0.001
AF258560	chr16:24930681-24930880	199	1.38	<0.001	0.005
LOXL2	chr8:23190561-23190780	219	1.35	<0.001	0.003
AC016644.1	chr5:56238121-56238320	199	1.35	<0.001	0.019
RP11-14N7.2	chr1:148934661-148934860	199	1.34	<0.001	0.006
AP001476.4	chr21:47470561-47470760	199	1.34	<0.001	0.020
RP3-399L15.2	chr6:114858501-114858700	199	1.31	<0.001	0.011
AK310441	chr1:148876821-148877060	239	1.28	<0.001	0.001
RP11-423O2.7	chr1:142958401-142958660	259	1.25	<0.001	0.017
LOC101928402	chrX:123095761-123095980	219	1.21	<0.001	0.008
LINC00521	chr14:94461821-94462080	259	1.18	<0.001	0.006
TBCE	chr1:235532261-235532520	259	1.16	<0.001	0.001
SMIM22	chr16:4838741-4839000	259	1.13	<0.001	0.003
XLOC_l2_000395	chr1:142839541-142839880	339	1.11	<0.001	0.026
SEMA4C	chr2:97531721-97531960	239	1.11	<0.001	0.012
LOC101929378	chr2:157111641-157111940	299	1.08	<0.001	0.010
ZNF169	chr9:97023241-97023440	199	1.07	<0.001	0.024
GNPTG	chr16:1409001-1409360	359	1.07	<0.001	0.004
TIMELESS	chr12:56816721-56817100	379	1.05	<0.001	0.002
RP13-638C3.3	chr17:80544641-80544940	299	1.02	<0.001	0.009
XLOC_009584	chr11:123084121-123084460	339	1	<0.001	0.006
**Hypomethylated**					
SSH1	chr12:109199901-109200160	259	−1.12	<0.001	0.004
XLOC_005639	chr6:21980601-21980860	259	−1.13	<0.001	0.025
RP11-458D21.1	chr1:145380441-145380780	339	−1.19	<0.001	0.011
LOC100506603	chr14:77252181-77252640	459	−1.19	<0.001	0.002
AK125727	chr14:77252181-77252640	459	−1.19	<0.001	0.002
AP001476.3	chr21:47477561-47477760	199	−1.24	<0.001	0.025
BC034416	chr3:180586661-180586880	219	−1.31	<0.001	0.007
RN7SL367P	chr16:1946361-1946700	339	−1.35	<0.001	0.004
RP11-586K12.4	chr16:32752701-32752900	199	−1.37	<0.001	0.005
EIF3B	chr7:2412041-2412260	219	−1.37	<0.001	0.004
ANKIB1	chr7:91999241-91999440	199	−1.37	<0.001	0.013
XLOC_010373	chr13:45618701-45618900	199	−1.38	<0.001	0.013
RP11-510M2.5	chr16:71577621-71577820	199	−1.38	<0.001	0.004
OPN1MW	chrX:153446941-153447140	199	−1.39	<0.001	0.008
ELAC2	chr17:12919641-12919840	199	−1.45	<0.001	0.012
SLC25A35	chr17:8196461-8196680	219	−1.52	<0.001	0.004
AK095057	chr5:179268841-179269080	239	−1.53	<0.001	0.024
C18orf8	chr18:21081741-21081940	199	−1.55	<0.001	0.002
RP11-168K11.3	chr9:116382121-116382460	339	−1.62	<0.001	0.005
LL22NC03-N27C7.1	chr22:24081461-24081680	219	−1.63	<0.001	0.009
CRAMP1L	chr16:1716841-1717040	199	−1.76	<0.001	0.014

Gene name refers to the name of the DMR-associated gene. Genomic coordinates refers to the genomic locus of the DMR. DMR Length refers to the length of the DMR. log2FC refers to the fold change of normalized tag counts between two groups (log2 transformed). The *p*-value refers to the *p*-value of the DMR, the smaller, the more significant. The *q*-value refers to the Benjamini-Hochberg False Discovery Rate (BH FDR) corrected *p*-value.
